# Progress towards a hepatitis C virus vaccine

**DOI:** 10.1038/emi.2013.79

**Published:** 2013-11-20

**Authors:** Lok Man John Law, Abdolamir Landi, Wendy C Magee, D Lorne Tyrrell, Michael Houghton

**Affiliations:** Li Ka Shing Institute of Virology, Department of Medical Microbiology and Immunology, University of Alberta, Edmonton T6G 2E1, Canada

**Keywords:** HCV, hepatitis, infection, prophylactic, therapeutic, vaccine

## Abstract

New drugs to treat hepatitis C are expected to be approved over the next few years which promise to cure nearly all patients. However, due to issues of expected drug resistance, suboptimal activity against diverse hepatitis C virus (HCV) genotypes and especially because of their extremely high cost, it is unlikely that these HCV drugs will substantially reduce the world's HCV carrier population of around 170 million in the near future or the estimated global incidence of millions of new HCV infections. For these reasons, there is an urgent need to develop a prophylactic HCV vaccine and also to determine if therapeutic vaccines can aid in the treatment of chronically infected patients. After much early pessimism on the prospects for an effective prophylactic HCV vaccine, our recent knowledge of immune correlates of protection combined with the demonstrated immunogenicity and protective animal efficacies of various HCV vaccine candidates now allows for realistic optimism. This review summarizes the current rationale and status of clinical and experimental HCV vaccine candidates based on the elicitation of cross-neutralizing antibodies and broad cellular immune responses to this highly diverse virus.

## HEPATITIS C AND THE NEED FOR A VACCINE

Hepatitis C is a major global health concern and the leading cause of liver transplantation in North America. The etiology of this blood borne disease is infection with the hepatitis C virus (HCV).^1^ Acute HCV infection causes mild disease and is usually asymptomatic. Most of the disease symptoms, including liver cirrhosis, are manifested during the chronic phase of infection which is life-long unless successfully treated. In some cases, infection with HCV can lead to the development of hepatocellular carcinoma. Currently, it is estimated that around 170 million people worldwide (∼2% of the population) are persistently infected with the virus and some of the chronic carriers are not aware of their infection.

The current standard of care therapy includes treatment with a combination of pegylated interferon (IFN) and ribavirin plus inhibitors of a viral-encoded serine protease. This therapy is about 70% effective in patients infected with genotype 1 virus, the most common genotype of HCV in the world. Infections with HCV genotypes 2 and 3 are currently treated with only interferon and ribavirin, and the overall treatment efficacy is about 80%. The regulatory approval of the protease inhibitors Telaprevir and Boceprevir marked the beginning of the clinical use of HCV-specific direct acting antivirals (DAAs).^2^ Many more DAAs are being developed against various virus-specific genes and proteins and will soon reach the clinic.^3^ A combination of these antiviral agents could present a possible treatment to overcome this infection in the future.

Despite this progress, many challenges remain. The current therapy is associated with many side effects. This leads to early termination of therapy in some cases resulting in suboptimal treatment. Further, genetic determinants in both the host and the virus can prevent 100% efficacy.^4,5^ Although the DAAs represent a step forward in the treatment of HCV, other problems occur with this therapy as well. Viral resistance against Telaprevir and Boceprevir has been observed clinically and has been associated with treatment failure.^6,7^ Importantly, the high costs of these new therapies and the large numbers of HCV-infected individuals means the health-care system, even in developed countries, cannot afford to treat all patients; this limitation is even more pronounced in developing countries. Therefore, development of a vaccine to prevent acute or chronic infection is essential. In this review, we summarize the strategies and progress on HCV vaccine development.

## VIROLOGY AND NATURAL IMMUNITY

HCV is an enveloped positive strand RNA virus of the family *Flaviviridae*.^8^ The virus genome encodes a large, single open reading frame that is subsequently processed into 10 major viral proteins. The first three are structural proteins: the nucleocapsid forming protein core, and the two envelope glycoproteins E1 and E2. The latter seven proteins are viroporin p7 and six non-structural (NS) proteins: NS2, NS3, NS4a, NS4b, NS5a and NS5b. HCV is a heterogeneous virus and is divided into seven major genotypes found worldwide. There is up to a 30% difference between these genotypes at the nucleotide level,^9^ especially in the region of the genome encoding structural proteins. Ideally, treatment for hepatitis C should be effective against all genotypes of the virus in order to confer global protection. However, the diversity of HCV translates into various sensitivities to treatment,^10,11^ none of which are 100% effective. The basic mechanism behind virus diversity lies with the viral RNA-dependent RNA polymerase (NS5b) which replicates the HCV genome. NS5b is an error-prone enzyme lacking proof-reading activity which results in HCV genome populations existing as a fluid RNA swarm termed quasispecies. This poses a problem for HCV treatment and vaccine development because resistance mutations within the virus population can potentially emerge and dominate under any therapeutic or vaccine selection pressure.

HCV has adapted exquisitely to escape host immune control with most cases progressing into a chronic or persistent infection. Nonetheless, 20% of acute HCV infections can be spontaneously cleared.^12^ Identifying the correlates of immune protection among these spontaneous resolvers is a central question for vaccine design and therapy. During primary HCV infection, the viral RNA accumulates in serum during the first 1–2 weeks. This is followed by induction of serum transaminase levels reflective of liver injury, which can be asymptomatic. At the peak serum transaminase level, adaptive T-cell responses to HCV can be detected.^13^ In the case of spontaneous virus clearance, this T-cell response is followed by a reduction of viremia to an undetectable level. Studies following individuals in this group of spontaneous resolvers showed that a broad virus-specific CD4^+^ and CD8^+^ T-cell response correlates with protection.^14,15^ In contrast, where infection progresses into a chronic state, adaptive immunity is weaker and ineffective in controlling the virus, HCV RNA remains high in serum and T cells show limited reactivity to HCV.^16,17,18^ The role of humoral immunity in HCV clearance is not well understood. It is thought that neutralizing antibodies are detected only in the chronic phase of infection.^19,20,21,22^ Although these antibodies cannot clear the infection, they do exert a selective pressure, driving virus evolution and suggesting immune regulation of the virus.^22,23^ Indeed, there is evidence that during the chronic phase of infection, HCV virions exist as immune complexes in sera bound to HCV-specific antibodies.^24^ With the recent development of a tissue culture system to study virus entry,^25,26,27,28^ the role of neutralizing antibodies is being better defined, particularly during the acute phase of infection.^29,30^ The role of antibodies in the control of HCV infection will be discussed in more detail below. Re-infection of individuals who spontaneously cleared their first HCV infection show reduced levels and duration of viremia compared to the primary infection. The rate of spontaneous clearance also rises from 20% to 80%.^29^ This increase in spontaneous clearance rate is suggestive of immune memory to HCV infection, which provides great encouragement for the effectiveness of a prophylactic vaccine.

## PROPHYLACTIC VACCINE

**Figure 1:**
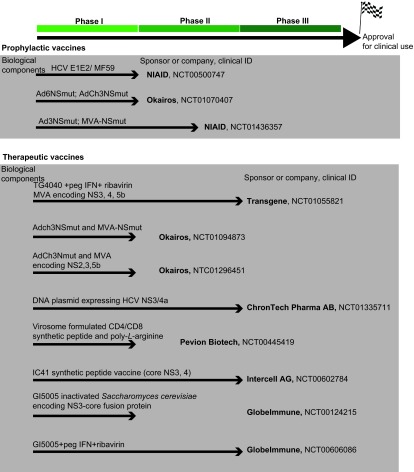
Summary of selected potential HCV vaccines in clinical development. These vaccines were grouped based on either prophylactic or therapeutic usage. They are currently either in phase I, phase I/II or phase II development (no HCV-specific vaccine has reached phase III development yet). The biological component(s) of the vaccine is listed on top of the arrow. Sponsor or company conducting the trial is listed at the end of arrow along with clinical ID number (http://www.clinicaltrials.gov). Selected examples of vaccines will be further discussed in the text. NIAID, National Institute of Allergy and Infectious Diseases.

There are two major approaches to a prophylactic vaccine for HCV. One is to target the envelope glycoproteins E1/E2 to induce neutralizing antibodies and the other is to target relatively conserved viral proteins within the non-structural region of the genome to induce a broad T-cell response (Figure 1). A meta-analysis of vaccine efficacy based on the chimpanzee model has suggested the importance of including structural protein regions in the vaccine.^31^ These data suggest that neutralizing antibodies might play a role in controlling infection.

**Figure 2:**
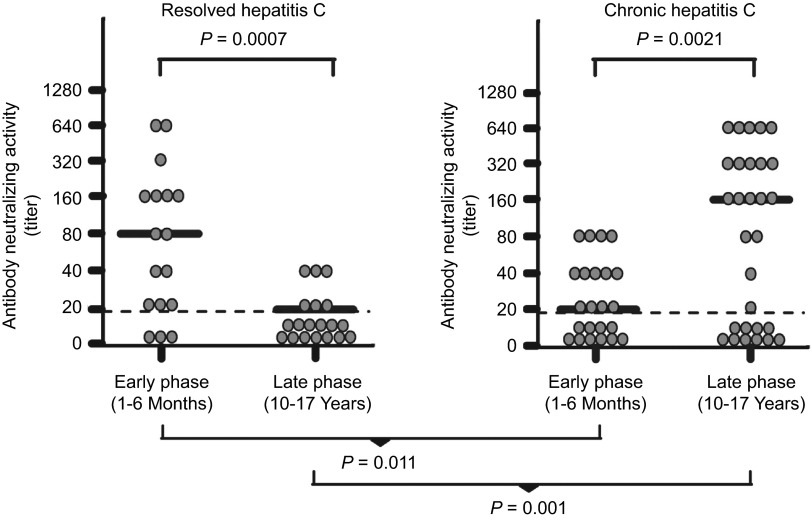
Neutralizing antibodies in patients with resolved or chronic hepatitis C. Anti-HCVpp neutralizing titers were determined by end point dilution of sera. HCVpp or control pp were pre-incubated for 1 h with serial serum dilutions before infection of Huh7 target cells. The end point titers of the early phase (1–6 months after infection) and late-phase (10–17 years after infection) serum samples are shown as scatter plots. The median titer is marked by a line. Data are expressed as means of two independent experiments performed in duplicate. Samples showing a titer of <1/20 were considered negative. The cutoff titer 1/20 is indicated by a dashed line. The data are reproduced with permission from Pestka *et al.*^30^ HCVpp, HCV pseudo-particles.

Most, if not all of the current successful vaccines in the market today are dependent on the induction of neutralizing antibodies to prevent or limit infection/disease.^32^ Even for another diverse virus such as HIV-1, the recent RV 144 human vaccine trial showed that modest reduction of infection risk is correlated with antibody response to the envelope spike of HIV-1.^33,34^ This approach to HCV vaccine development is strengthened by recent studies of HCV infection showing that neutralizing antibodies correlate with HCV clearance.^29,30,35^ In a single-source outbreak of HCV, Pestka *et al.*^30^ showed that patients who resolve HCV infection have a higher level of neutralizing antibodies during the acute phase infection (Figure 2). Response to interferon therapy has also been correlated with antibody titers to virion proteins.^36^ One vaccine designed to induce a humoral response against HCV used recombinant glycoprotein (gp) E1/gpE2 adjuvanted with MF59. In the chimpanzee model, this vaccine showed efficacy in reducing the rate of chronicity following both homologous and heterologous 1a virus challenge.^37^ In a few cases, it also completely prevented infection against homologous challenge.^37^ Importantly, if vaccination can reduce the incidence of chronic infection, it will be very effective since HCV-associated disease is manifested mostly during this chronic phase. The safety and immunogenicity of this recombinant glycoprotein-based vaccine has been tested in humans in a phase I clinical trial. The results of this trial indicated that the vaccine induced strong humoral and CD4^+^ T-cell responses and the vaccinated volunteers presented with minimal side effects.^38^

The major drawback of this vaccine approach is the heterogeneity of HCV as described earlier. Historically, neutralizing antibodies were presumed to be genotype-specific rendering it very difficult to confer global protection.^21,39,40,41^ However, other studies describing broadly cross-neutralizing antibodies that prevent infection have been reported in the literature.^42^ Most of these antibodies recognize conserved regions mainly within the glycoprotein E2, although some recognizing E1 have also been described.^43^ Some of these cross-neutralizing antibodies target discontinuous epitopes suggesting conformation-dependent recognition. It is possible that the virion envelope glycoproteins from the various genotypes maintain a conserved globular structure in order to interact with the conserved entry pathway, despite substantial genomic diversity at the primary sequence level.^44^ Glycoprotein based vaccination has been shown to induce cross-genotype neutralizing activity in chimpanzees and humans.^45,46^ Recently, our group showed this same vaccine induced broad neutralizing antibodies against representatives of all seven major HCV clades from around the world, although with varying efficacy.^47^ Whether this vaccine induces similar cross-neutralizing B-cell epitopes as those previously reported is currently being investigated. Furthermore, it has been reported that HCV can spread from cell to cell in order to avoid neutralizing antibodies.^48^ Whether vaccine-induced antibodies can prevent this mode of transmission is an open question although certain antibodies capable of preventing this mode of transmission have been reported.^49,50^

An alternative method to produce vaccines designed to elicit neutralizing antibodies is the use of whole, killed virus. This method is used in many licensed vaccines such as influenza A, hepatitis A, polio, rabies, Japanese encephalitis and papilloma virus vaccines.^51^ This approach was recently tested for HCV by Akazawa *et al.*^52^ Inactivated cell culture-derived HCV virions are capable of inducing cross-genotype neutralizing antibodies and confer protection against HCV infection in a mouse model by passive immunization with vaccinees' serum. This work opens up an important new avenue for HCV vaccine development. However, regulatory approval of a prophylactic vaccine produced in the transformed hepatocyte Huh7 cell line may be difficult. Another approved cell line for virus propagation may be required and these cell lines would need to be modified in order to support HCV particle production so that they express virus entry receptors, microRNA 122 and the apolipoproteins essential for virus assembly.^53,54^ In addition, the low yield of HCV particle production in cell culture could limit its widespread use. The recent demonstration of much higher viral yields by culturing HCV-producing cells in the presence of human serum (rather than calf serum) could overcome this latter obstacle.^55^ Virus-like particles presenting HCV envelope proteins to induce neutralizing antibody is an alternate approach aiming to improve safety and low yield. Garrone *et al.*^56^ has reported HCV virus-like particles capable of inducing cross-neutralizing antibodies in an animal model. This result provides an encouraging result for further clinical testing. However, since HCV glycoproteins are arranged differently on virus-like particles than on the native HCV virion, the breadth of the subsequently induced antibodies should be compared.

**Figure 3:**
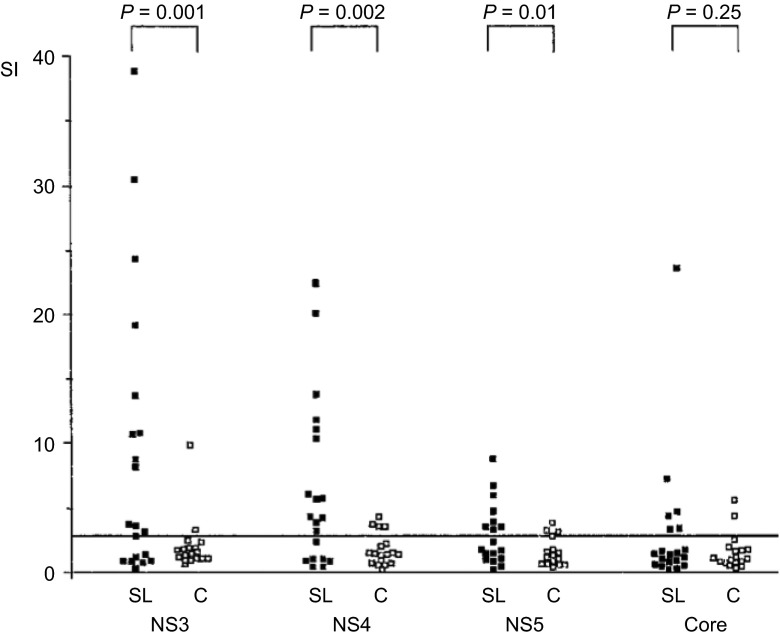
Proliferative CD4^+^ T-cell response of the first sample in the acute phase of disease to recombinant HCV proteins (HCV-NS3, -NS4, -NS5 and -core) of PBMCs from 38 patients with acute hepatitis C. Patients are grouped according to the final outcome of disease in self-limited hepatitis C (SL, *n*=20) and patients with chronic evolution (C, *n*=18). Results are shown as SI=^3^H-thymidine incorporation of antigen-stimulated PBMCs (counts per minute)/unstimulated control. All patients with self-limited disease displayed a significant proliferative T-cell response against at least one of the viral proteins, while patients with chronic evolution mounted no or only transient antiviral T-cell responses. NS3 and NS4 revealed the most frequent and most vigorous responses. In four patients, the proliferative response against NS5 was not tested in the first sample. The data are reproduced with permission from Gerlach *et al.*^57^ PBMC, peripheral blood mononucleated cell; SI, simulation index.

Another HCV vaccine approach aims at induction of broad cellular immunity using the delivery of HCV genomic regions encoding the non-structural proteins. Spontaneous resolvers of acute HCV infection have been shown to elicit strong, broad HCV-specific cellular immune responses, whereas individuals progressing to chronic, persistent infection exhibit much weaker and narrowly-targeted cellular immune responses (Figure 3).^57^ Further, immunodepletion of either CD4^+^ or CD8^+^ T cells in the chimpanzee HCV infection model leads to a progression to chronic infection confirming the importance of T cell-mediated immunity in preventing chronicity.^14,15^ Furthermore, eradication of HCV in chimpanzees can still occur in the absence of antibodies against gpE1/gpE2.^58,59,60^ Since the region encoding the non-structural proteins is less diverse than the structural protein-encoding region, this approach provides an attractive advantage compared to the glycoprotein approach. Okarios Inc. has tested the delivery of the HCV NS3, NS4a, NS4b, NS5a and NS5b genes of genotype 1b using a combination of replication-defective modified vaccinia Ankara and chimpanzee-derived adenovirus 3 vectors.^61^ Efficacy has been demonstrated by showing suppression of acute viremia and acute hepatitis after heterologous genotype 1a virus challenge in chimpanzees vaccinated with the prototype vaccine.^62^ However, no significant reduction in the chronic carrier rates was observed, possibly due to the small numbers of animals tested. Currently, this vaccine is being tested for efficacy in intravenous drug users in the United States and this trial is expected to be completed by 2015/2016.^63^

It would be pertinent in future to test the combination of envelope glycoprotein-based and T cell-based vaccines for potential additive or synergistic effects to boost efficacy. A recent study that followed spontaneous clearance of chronic HCV infection highlighted the role of both the humoral response as well as T-cell immunity.^35^ Mechanistically, it is probable the cross-neutralizing antibodies could limit the acute infection, which then allows effective T-cell immunity to efficiently clear the infection.

## THERAPEUTIC VACCINE

There are many mechanisms leading to the dysfunction of HCV-specific T cells, thus rendering them ineffective in the control of infection.^13^ Reactivation of these HCV-specific T cells is critical for a therapeutic vaccine to induce recruitment to the liver where they can exert their antiviral activity by secreting cytokines such as IFN-γ and tumor necrosis factor-alpha (TNF-α) and by direct killing of infected hepatocytes. The efficacy of these vaccines may possibly be improved by prior treatment with DAAs to first suppress HCV viremia (Figure 1).

One therapeutic vaccine candidate was codeveloped by CSL Limited and Chiron Corporation based on recombinant HCV core formulated with the T-cell adjuvant IMX. This vaccine demonstrated encouraging animal data^64^ and favorable phase I trial data in healthy human volunteers.^65^ Preliminary results also showed modest reduction in the viral load in a subset of chronic HCV patients.^37^ Transgene Inc. has used a modified vaccinia Ankara vector expressing the HCV NS3, NS4a, NS5a and NS5b genes to boost CD4^+^ T helper and CD8^+^ cytolytic T cells against these antigens. In a phase I clinical trial, 6 of 15 treated patients showed a reduction (0.5–1.4 log) in viral load following vaccination. The two patients with the highest reduction in virus titers also showed a concomitant increase in vaccine-specific T-cell responses.^66^ Recently, Transgene's report of a phase II clinical trial showed that pre-treatment of HCV-infected patients with the vaccine prior to treatment with INF-α and ribavirin increased the early virological response (64% *versus* 30% in the control group).^67^ However, significant side effects of this combined therapy have been reported rendering this regimen potentially problematic. Okarios is conducting a phase Ib trial using the prime/boost method with replication defective adenovirus 6 and modified vaccinia Ankara expressing the HCV NS3, NS4a, NS4b, NS5a and NS5b genes in concert with standard-of-care drug therapy.^68^ Another approach was used by the Swedish company ChronTech to directly deliver a DNA plasmid encoding the HCV proteins 3/4a by electroporation. This vaccine has currently moved into phase II clinical testing.^69^

It will be of interest to determine the efficacy of these therapeutic vaccines in combination with interferon-free DAA therapy. However, the probability of future combinations of DAAs being able to cure all HCV patients is so high that developing therapeutic vaccination strategies for HCV may be unnecessary.

## PERSPECTIVE

Combinations of new HCV DAAs to effectively treat chronic HCV infections are expected to become available over the next 1–2 years. However, it is very unlikely that these very costly drug combinations can be made accessible to most HCV carriers around the globe since treatment of all of these individuals will cost in the region of US$10 trillion! Therefore, we consider the development of a global prophylactic vaccine to be of high priority.

The rate of liver cancer is rapidly climbing^70^ and one of the major risk factors is HCV infection. Many individuals were unknowingly infected with HCV prior to the identification of the virus about 25 years ago. Since many of these infections are only now manifesting as late stage liver disease, the incidence of HCV-related morbidity and mortality will continue to climb in the future.^71,72,73^ Therapeutic vaccines could provide a needed boost to complement the success of HCV DAAs to combat chronic infection. However, these vaccines are aimed at boosting HCV-specific T cells targeting infected liver cells. As such, there is a risk that these could potentially increase liver injury and exacerbate inflammation within the liver of these chronic HCV carriers. Continual monitoring of the safety of these therapeutic vaccines will be critical. Their use is also likely to be limited unless they can be provided at much lower cost than HCV DAAs.

Recently, some genetic factors have been identified that favor the outcome of HCV therapy.^4,5,74,75,76^ In particular, IL28B polymorphisms have been linked to spontaneous clearance as well as to a favorable response to IFN-α based therapy.^4,5,74,75^ It is not yet known if vaccine efficacy will be similarly tied to host genetics. Expansion of the population tested with promising vaccine candidates will help to answer these questions. Further exploration of the immune correlates of HCV clearance will also aid in improving vaccine design and regulatory approval. Additional cohorts of patients are currently being followed during acute HCV infection in order to answer these questions.

With the arrival of many more HCV-specific DAAs and promising candidates for HCV vaccine antigens and delivery on the prophylactic and therapeutic fronts, HCV therapy options are rapidly expanding. The obstacle to HCV prevention and treatment will soon be an economic and political issue in terms of how to effectively divide health-care resources for HCV therapy and prophylactic vaccine implementation.
